# Plant viral intergenic DNA sequence repeats with transcription enhancing activity

**DOI:** 10.1186/1743-422X-2-16

**Published:** 2005-02-24

**Authors:** Jeff Velten, Kevin J Morey, Christopher I Cazzonelli

**Affiliations:** 1 USDA-ARS, Plant Stress and Water Conservation Laboratory, 3810 4th St., Lubbock, TX 79415, USA; 2 Department of Biology, Colorado State University, Fort Collins, CO 80523, USA

## Abstract

**Background:**

The geminivirus and nanovirus families of DNA plant viruses have proved to be a fertile source of viral genomic sequences, clearly demonstrated by the large number of sequence entries within public DNA sequence databases. Due to considerable conservation in genome organization, these viruses contain easily identifiable intergenic regions that have been found to contain multiple DNA sequence elements important to viral replication and gene regulation. As a first step in a broad screen of geminivirus and nanovirus intergenic sequences for DNA segments important in controlling viral gene expression, we have 'mined' a large set of viral intergenic regions for transcriptional enhancers. Viral sequences that are found to act as enhancers of transcription in plants are likely to contribute to viral gene activity during infection.

**Results:**

DNA sequences from the intergenic regions of 29 geminiviruses or nanoviruses were scanned for repeated sequence elements to be tested for transcription enhancing activity. 105 elements were identified and placed immediately upstream from a minimal plant-functional promoter fused to an intron-containing luciferase reporter gene. Transient luciferase activity was measured within *Agrobacteria*-infused *Nicotiana tobacum *leaf tissue. Of the 105 elements tested, 14 were found to reproducibly elevate reporter gene activity (>25% increase over that from the minimal promoter-reporter construct, p < 0.05), while 91 elements failed to increase luciferase activity. A previously described "conserved late element" (CLE) was identified within tested repeats from 5 different viral species was found to have intrinsic enhancer activity in the absence of viral gene products. The remaining 9 active elements have not been previously demonstrated to act as functional promoter components.

**Conclusion:**

Biological significance for the active DNA elements identified is supported by repeated isolation of a previously defined viral element (CLE), and the finding that two of three viral enhancer elements examined were markedly enriched within both geminivirus sequences and within *Arabidopsis *promoter regions. These data provide a useful starting point for virologists interested in undertaking more detailed analysis of geminiviral promoter function.

## Background

Traditionally, analyses of viral promoter structure-function relationship have involved directed deletion or disruption of promoter structure, followed by determination of resulting changes in transcription, if any, resulting from the alterations [1]. A relatively small subset of the promoter elements identified in this way have been subsequently isolated and tested for their ability to influence transcription when inserted into alternative, well defined, basal promoters [2]. As an alternative to so-called 'promoter bashing' approaches to the study of promoter structure, we have instead chosen to 'mine' specific regions of viral DNA for sequence elements that, when combined with a minimal plant promoter, are able to enhance transcription of a reporter gene *in planta*.

To test the enhancer mining approach we chose to examine a collection of geminivirus and nanovirus intergenic sequences obtained from GenBank. There are a relatively large number of available sequences for these DNA viruses and due to conserved genomic organization they contain easily identifiable intergenic regions [3]. Additionally, several studies have demonstrated *in planta *promoter activity using isolated or modified geminivirus or nanovirus intergenic sequences [4-21]. Although some areas of sequence similarity exist within the intergenic regions of the geminiviruses [22], very few of these common sequence elements have been experimentally shown to contribute to transcriptional activity. We specifically avoided using any test for evolutionary conservation of candidate elements, hoping to identify unique elements that may not necessarily be shared by large groups of related viruses. For this first broad screen, the experimental rational used made two basic assumptions; 1} that viral intergenic regions contain an enrichment of DNA transcriptional regulatory elements; and 2} that important regulatory sequence elements are often duplicated within promoters, either directly repeated, or as inverted copies of sequence segments [22].

The described enhancer mining of viral sequences is not intended to be a comprehensive analysis of viral promoter structure since by design it is limited to identification of promoter elements that up-regulate gene expression and that make use of endogenous plant transcription factors available within the un-infected test plant. However, based upon their iteration, location within intergenic regions, and ability to enhance transcription *in planta*, any elements identified using this approach are likely to contribute to regulation of *in vivo *viral gene expression during plant infection. By allowing relatively large numbers of viral sequences to be examined using a defined system, the approach has the potential of generating data useful in comparing positively acting viral promoter elements within and between viral families. In addition, identification of elements that are active *in planta *in the absence of viral infection provides results pertinent to understanding virus-host interactions at the level of gene control. Finally, the resulting list of active and inactive viral sequences provides a valuable starting points for subsequent, more detailed, analysis of transcription regulation of individual viruses.

## Results

### Search for candidate elements

**Figure 1:**
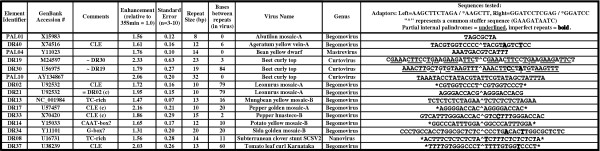
**Viral enhancer elements. **All viral repeats that produced greater than a 25% increase in 35S min activity are listed. For each active element the accession number, relative enhancement (with standard error), repeat length, repeat separation, source virus (and genus) and viral sequence are shown. Adaptor sequences are listed in the header of the sequence column and with imperfect repeats in bold and partial palindromes within repeats underlined.

The initial search for sequence repeats was performed on the major intergenic regions of 29 different geminivirus or nanovirus genomic sequences (Figure 1 and Additional file 1). The search was arbitrarily halted after 105 candidate repeats were identified and was not intended to provide a comprehensive representation of all duplicated sequences within any of the viral sequences examined. Although generated using different search criteria than those employed by Arguello-Astorga *et al *[22], the resulting collection of geminivirus sequence repeats contains some sequences similar or identical to the described "iterons" (it should be noted that functional testing of nearly all of the "iterons" listed has not yet been reported in the literature).

### Functional testing of elements

Of the 105 repeats tested (Figure 1 and Additional file 1), 14 (13%) reproducibly resulted in increases of at least 25% above that of the 35S min construct (p < 5% by Student's T-test, the T-test was used only as a guide since by the nature of the assay used, individual data sets are small) (Figure 1 and Additional file 1). The remaining 91 (87%) failed to produce any measurable enhancement of reporter gene activity (see Additional file 1). All the positive elements identified by the *in vivo *assay were subsequently tested using an *in vitro *dual-luciferase^® ^system from Promega Corp. and produced levels of enhancement very similar to those obtained using the *in vivo *assay (the enhancement values and standard error reported in Figure 1 and Additional file 1 include both *in vivo *and *in vitro *data normalized to 35S min = 1.0). The observed enhancement of promoter activity (~2 fold) is relatively modest compared to other viral transcriptional enhancers that have been isolated and tested (e.g., G-box [23] and AS-1 [24] type elements enhance 35S min activity 8–10 fold using this assay, data not shown). This outcome may reflect limitations of the original search parameters (only repeated elements were tested). However, several of the geminiviral elements identified in this screen have been subsequently found to display clear and unique synergistic effects when combined or multimerized (Cazzonelli, Burke and Velten, manuscript in preparation), supporting their potential to contribute to viral gene regulation during infection.

Since all assays were performed on tobacco plants that had been neither infected with any of the viruses screened, nor transfected with any viral components, it is unlikely that elements strictly dependent upon virally encoded regulatory factors, or factors not native to *N. tobacum*, would be identified. In addition, the screen was limited to those elements that increase gene expression, and no effort was made to confirm data suggesting that an element might be a 'repressor' (e.g., the 11 elements that show 'enhancement' values less than, or equal to, one third of the 35S min activity, see Additional file 1). Considering these limitations, the finding that 13% of the sequences tested produced measurable up-regulation of transcription supports the original assumption that basic transcription regulatory elements are enriched within repeated sequences from the viral intergenic regions. Despite having tested approximately equal numbers of inverted sequence repeats (IR) and direct sequence repeats (DR), 11 of 14 active elements were members of the DR set, with the remaining 3 positives being palindromic (inverted repeats with no sequence between the repeats). This is somewhat surprising since many of the iterated DNA sequence elements within geminivirus intergenic regions are found as both direct and inverted repeats [22], and as such could have been present in either the DR or IR set of elements. Although the numbers tested are small, and the screen was performed using a single plant species, these results suggest that directly repeated sequences within geminivirus and nanovirus intergenic repeats have a higher probability of positively influencing transcription levels than do the inverted sequence structures. It is possible that this bias may reflect the presence within the intergenic region of DNA elements responsible for viral replication [25], including a conserved inverted repeat structure with a ubiquitous central-loop sequence [26]. Seven of the IR elements tested in this study are part of predicted replication hairpin structures (see Additional file 1) and did not, in this test system, result in any measurable enhancement of reporter gene expression.

**Figure 3:**
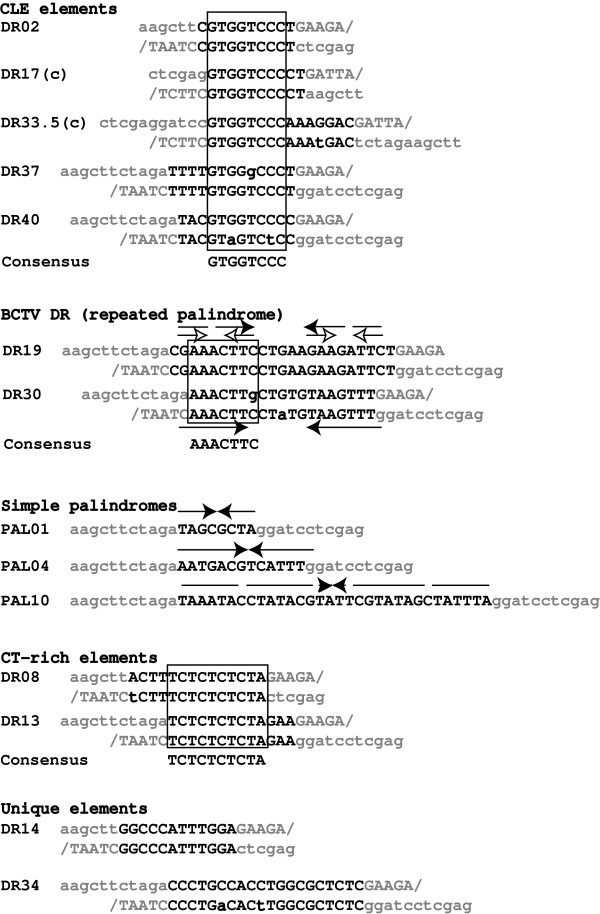
**Alignment of active repeat elements. **Each directly repeated element is offset (at the "/") to align both copies of the repeat. Related elements are additionally aligned as paired repeat alignments. Bases that differ within paired repeats are in lowercase bold and palindromic sub-elements within the repeats are indicated by arrows. Areas of the alignments used to determine a consensus sequence are boxed.

Manual alignment of all the active DR sequences produced three classes of related elements and several unique individuals (Figure 3). Five of the 14 positive DR elements contain an already identified geminiviral transcription control element, the "conserved late element" or CLE {GTGGTCCC, [22,27]}. The CLE sequence had been previously shown to affect expression from a minimal 35S promoter, and to be up-regulated by the viral AC2 gene product [27]. The two remaining grouped elements include a pair of "CT" rich repeats (DR08 and DR13) and two related, nearly-palindromic direct repeats from beet curly top virus (BCTV, elements DR19 and DR30). Despite the lack of an exact G-box core sequence {ACGT, [28]}, the nearly palindromic structure of the DR19 and DR30 elements {aaACTTc} is reminiscent of duplicated G-box type geminiviral elements noted by Arguello-Astorga et al [22] and later proposed as functional components within tomato golden mosaic virus (TGMV) and subterranean clover stunt virus (SCSV) promoters [11,20]. When scanned against the online PlantCARE promoter element database {[29,30]} no clear consensus emerges regarding similarity of the discovered viral elements with characterized plant cis regulatory elements (the most common hits were against light or stress responsive elements, although that may simply represent the distribution of plant elements contained within the database).

### Element occurrence in viral and *Arabidopisis *sequence databases

Short of directed mutagenesis of each identified viral element, followed by analysis of resulting 'mutant' virus function within infected plants, it is difficult to directly determine what contribution each of the identified enhancer elements makes to viral gene regulation. Computer analysis of an element's frequency of occurrence in defined DNA sequence databases provides an alternative mechanism for gaining insight into likely biological function for short sequence elements [31]. For example, the occurrence frequency of functionally important promoter elements is higher within DNA sequences upstream from gene coding regions, compared to the frequency within non-regulatory sequences [31]. Since the element enrichment approach works best when applied to relatively short, core consensus sequences [31], viral element searches were limited to those viral enhancers that showed a clear core consensus (CLE, BCTV DR19/30, CT-rich, Figure 3).

The viral enhancers identified in this work were found to function within un-infected test plants, indicating that the viral elements can make use of intrinsic plant transcription factors (not virally encoded) and may, therefore, be similar or identical to endogenous plant promoter elements. In order to test for enhancement of viral enhancer sequences within higher plant promoters, the PatMatch page of the TAIR web site [32] was used to access sub-datasets of the *A. thaliana *genomic sequence that are exclusive to annotated coding sequences {CDS} and three upstream sequence lengths {-3000, -1000, -500 bp, measured from each CDS start codon}. Each of the sub-datasets was searched for the viral elements (CLE, BCTV DR19/30, CT-rich) and, as controls, several well defined plant promoter element consensus sequences (the "G-Box" {CACGTG}, a common plant promoter element that is associated with members of the pZIP family of transcription factors [33,34], and two less prevalent plant promoter elements, the drought response element ('DRE', RCCGAC [35]) and abscisic acid response element (ABRE-like, ACGTGKM) [35]).

Performing similar oligonucleotide frequency searches for element enrichment within viral promoters was complicated by the lack of comprehensive annotation of viral sequence entries within the GenBank database. Without clear annotation of intergenic and coding sequences within the viral GenBank entries, it was impossible to directly perform the same sort of 'upstream sequence' (in this case, viral intergenic regions) versus 'coding sequence' frequency comparisons that were possible using the fully annotated *Arabidopsis *genome sequence and PatMatch. As an alternative, screens were performed to determine frequencies of occurrence for viral enhancers (and control plant elements) within a sequence database consisting of all geminivirus or nanovirus GenBank entries as of May 13, 2004 [36], and the results compared with those obtained scanning the same sequences against the *Arabidopsis *PatMatch datasets. The searched viral sequence database has the potential for bias due to the existence of a numerous entries containing only coding regions or only intergenic sequences, as well as some duplication of sequences in separate entries. Any such bias should, however, similarly affect the baseline frequency values resulting from searches using the 18 matched random oligonucleotides (in parenthesis, Table 1), thus all element enrichments are considered relative to the random oligo values. It was decided to perform the searches using the full geminiviral plus nanoviral database, since limiting the viral entries to only those containing fully annotated, complete viral sequences would have greatly reduced the number of different viruses examined.

**Table 1:** Element occurrence frequencies within viral and *Arabidopsis *sequence databases

**Element Identifier**	**Element Sequence**	**Occurrence frequency from each database. Values are relative to *Arabidopsis *CDS = 1.00 (Mean of 18 matched oligomer frequencies)**				
		***Arabidopsis *-3000 to -1001**	***Arabidopsis *-1000 to -501**	***Arabidopsis *-500 to -1**	***Arabidopsis *CDS**	**Gemini + nanovirus**

Previously Identified Promoter Elements from *A. thaliana *(*also confirmed as geminiviral element)						

ABRE-like	ACGTGKM	1.65 (1.72)	1.78 (1.8)	**3 **(1.44)	1 (1.59)	**2.45 **(1.28)
DRE	RCCGAC	1.86 (1.75)	1.81 (1.55)	2.13 (1.46)	1 (1.13)	1.09 (0.85)
G-box*	CACGTG	2.28 (1.79)	2.57 (1.58)	**4.35 **(1.47)	1 (1.41)	**3.81 **(1.43)

Consensus Gemini/Nanoviral Sequence Elements (**not a promoter element)						

CLE	GTGGNCCC	3.15 (3.51)	3.62 (2.99)	**3.9 **(2.79)	1 (1.56)	**17.36 **(2.81)
DR08/13	TCTCTCTCTA	**3.15 **(0.46)	**3.6 **(0.4)	**7.75 **(0.35)	1 (0.51)	**6.92 **(0.53)
BCTV DR19/30	AAACTTC	0.7 (0.62)	0.69 (0.64)	0.68 (0.66)	1 (0.72)	0.64 (0.52)
GV rep-stem**	CGCGNCCA	2.52 (3.51)	2.2 (2.99)	2.26 (2.79)	1 (1.89)	**17.11 **(3.42)

The results of the searches are displayed in Table 1. Each frequency value (cHits/Mbp) represents the number of hits per million base pairs, corrected for the database base composition using empirically determined G/C and A/T ratios for each of the databases examined (see Materials and Methods). To facilitate comparison, the resulting cHits/Mbp from the *Arabidopsis *upstream databases (-3000 to -1001, -1000 to -501, and -500 to -1 bp) were normalized relative to the value obtained for each element's occurrence within the *A. thaliana *coding sequence database (CDS value set to 1.0). In addition to the predicted frequency values, in each case, the element's observed frequency was also compared to a value generated using the average of 18 random oligomers having the same length and base composition as the element tested (in parenthesis, Table 1). The test sequences for plant ABRE-like and G-box elements showed clear enrichment within the upstream *Arabidopsis *sequences, especially within the -1 to -500 region (ABRE-like element = 3.0 time the CDS value, vs 1.44 for random sequences and G-box = 4.35 vs 1.47 for random sequences, all as normalized cHits/Mbp). Results for the DRE element were less convincing (2.13 vs 1.46 in the -1 to -500 dataset) and likely reflect lower functional usage of this element within the *Arabidopsis *genome [35].

As expected, the CLE consensus sequence (GTGGNCCC) was found to be markedly enriched within the viral database, occurring 6 times more frequently than the mean of 18 random 8-mers of identical base composition (CLE = 17.36 normalized cHits/Mbp vs 2.81 from matched random sequences). This frequency is similar to that found (17.11 vs 3.42) using a short sequence of identical base composition and length that matches a highly conserved replication stem-loop sequence (CGCGNCCA), a component that is evolutionarily conserved within the geminivirus population [37]. Enhancement of CLE within *Arabidopsis *promoters is less obvious (CLE = 3.9 in the -1 to -500 database vs 2.79 for random sequences). The observed relatively small CLE enrichment is consistent with reports of a low frequency of occurrence for a CLE-like "TCP domain" binding consensus sequence (Gt/cGGNCCC) within *Arabidopsis *promoters [38]. It is possible that TCP domain-containing transcription factors contribute to the observed CLE enhancer activity since *Arabidopsis *promoters containing the TCP domain consensus binding element were found to function in transgenic tobacco and to show reduced activity after mutation of the element's core sequence [38].

The test sequences for plant element occurrence within the viral database (ABRE-like = 2.4 vs 1.28 and G-box = 3.81 vs 1.43, DRE = 1.09 vs 0.85) provide further indication of the technique's utility. The G-box viral frequency is consistent with a previous report that a G-box element contributes to transcriptional regulation from the major intergenic region of Tomato Golden Mosaic Virus {TGMV, ([20]}. The ABRE-like element enrichment in the viral database may indicate that viruses make use of biotic and abiotic stress-induced up-regulation [39] of genes driven by ABRE-containing promoters, a possibility open to additional research.

Of the remaining viral elements tested against the *Arabidopsis *and viral databases (Table 1), only the DR08/13 TC-rich sequence showed clear enrichment in both plant promoter and viral sequences (*Arabidopsis *-1 to -500 = 7.75 vs 0.35 and viral = 6.92 vs 0.53). Similar TC-rich regions have been reported within plant promoter regions [40,41], but we are unaware of any published report that confirms enhancer activity associated with an isolated TC-rich element, either viral or plant in origin.

## Discussion

Except for the CLE elements, none of the active elements identified in this work have been experimentally reported as regulatory components of viral promoters. This is likely a reflection of both the limited number of geminivirus and nanovirus promoters that have been examined in detail [4,5,11,12,14,20,27,42,43], and the alternative approach of examining individual isolated elements used in this study. The mapped promoter components within the intergenic region of Tomato golden mosaic virus (TGMV) sub-genome A (TGMV-A) [14,20] provide a useful benchmark for comparison of results from this enhancer screen. Application of the repeated sequence screen to the TGMV (component B) intergenic region identified a single TGMV Direct repeat, DR38, and a single palindrome (PAL20), both of which were found to be inactive in our assay. This is consistent with published work that indicates most of the defined regulatory sequences within the TGMV-A intergenic region appear to occur as single copies [14,20]. The screen of intergenic repeats reported in this paper did, however, identify the CLE element, one copy of which has been shown to be part of the TGMV-A rightward promoter [14,20]. It is clear that testing only repeated elements will not identify all components of a promoter region, and when focusing on a specific promoter, testing of non-repeated elements (perhaps identified by evolutionary conservation) should be combined with other techniques such as insertion scanning [44]. Recently a collection of plant-functional promoters and terminators were isolated from the set of 7 Subterranean clover stunt virus (SCSV1-SCSV7) sub-genomic circles. The collection of sequence repeats tested in this study included 11 inverted or direct repeats from SCSV circles, only one of which (DR08 from SCSV2) showed any enhancing activity. It will be interesting to see how these tested repeated elements behave when examined in the context of the remainder of the SCSV promoter components.

## Conclusion

This screen of viral intergenic repeats was undertaken to specifically identify general transcriptional enhancing elements contained within intergenic regions of a subset of geminivirus and nanovirus genomes. The screen was successful in demonstrating transcriptional enhancer activity from one proven viral promoter element and several previously unidentified elements. The occurrence of the repeated elements within intergenic regions, combined with the clear enrichment within viral sequences and *Arabidopsis *upstream sequences for at least the CLE and TC-rich (DR08/13) classes of elements, strongly supports participation of the enhancers in viral gene expression.

The technique of testing isolated elements represents an alternative to normal promoter-by-promoter dissection and provides a useful tool for screening promoter regions for potential functional elements that have been implicated by any number of possible criteria (e.g. copy number, evolutionary conservation, comparison of promoters with similar function, microarray data, etc.). Although the number of elements tested is relatively small and, so far, only representative of promoters from the geminiviruses and nanoviruses classes of plant viruses, there is a clear trend suggesting that directly repeated elements (including those containing small internal palindromic sequences) are more likely to play significant roles in the enhancement of transcription than inverted repeats. This work represents one of the first attempts to directly screen for individual plant promoter elements that are isolated from their native promoter context. It is therefore, difficult to gauge the actual contribution of any of the elements identified to viral gene regulation and biological activity. These results do, however, provide a useful starting point for more detailed analyses of not only geminivirus and nanovirus promoters, but also overall plant promoter structure-function relationships.

## Methods

### Identification of sequence repeats

The search for repeated DNA sequences was performed by visual inspection of computer-generated dot matrix comparisons (criteria: ≥ 66% identity, 10 base window, GeneWorks v2.5.2, Oxford Molecular Group Inc.). Dot matrices generated using each viral plus strand plotted against itself were used to identify direct repeats while inverted repeats were found by plotting each plus strand against its complement.

### Production of sequence repeat test constructs

**Figure 2:**
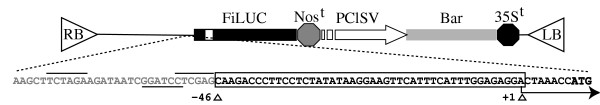
**T-DNA map of plasmid 35S min (in pPZP212). **T-DNA borders: RB = right border, LB = left border, FiLUC = firefly luciferase, Nos^t ^= nopaline synthase transcription terminator, PClSV = Peanut chlorotic streak virus promoter, Bar = phosphinothricin acetyl transferase, 35S^t ^= transcription terminator for the Cauliflower mosaic virus (CaMV) 35S transcript. DNA sequence insert shows the minimal 35S promoter from CaMV, from -46 to +1 (transcription start). Upstream from the minimal 35S promoter are the restriction sites (underlined: HindIII; BamH, overlined: XbaI; KpnI) used to insert test sequences and downstream is the start codon from the luciferase coding region (bold ATG).

The identified repeats were synthesized as DNA cassettes containing the duplicated elements in their original orientation, either directly repeated with spacer sequence ('DR', 41 elements), inversely repeated with spacer sequence ('IR', 45 elements), or palindromic inverted repeats without spacer ('PAL', 20 elements). In order to limit the tested component to only the repeated elements themselves, any sequence occurring between the viral repeats (ranging from 0 to 146 bp, median separation = 9 bp) was replaced with a 10 bp randomized stuffer sequence (GAAGATAATC). The resulting cassettes were inserted immediately upstream from a minimal promoter (-46 to +1 relative to transcription start, 35S min) reporter system derived from the cauliflower mosaic virus (CaMV) 35S promoter fused to an intron-modified firefly luciferase (FiLUC) gene (Figure 2, [45]). The resulting test constructs were generated as part of a modified pPZP211 [46] binary plant transformation vector (Figure 2) and were introduced into the *Agrobacteria tumefaciens *strain, EHA105 [47] by electroporation [48]. The final *Agrobacteria *strains each contain, in addition to the test plasmids, a second, compatible, binary transformation vector expressing an intron-modified version of the *Renilla reniformis *luciferase gene (RiLUC) [49] under control of the constitutive Super-promoter [50]. The FiLUC and RiLUC enzymes can be independently assayed, making the co-transferred constitutive RiLUC gene a useful marker for gene transfer and for normalization of FiLUC values between individual elements [45].

### Lucifrease assays

*Agrobacteria *harboring the test and normalization binary plasmids were grown at 28°C in LB media containing the appropriate antibiotic selection (25 μg/mL kanamycin sulfate or 100 μg/mL spectinomycin) until an OD_600 _of 0.8 was achieved. The resulting cultures were centrifuged at 3000 rpm for 15 minutes, washed and re-suspended in an equal volume of infiltration media (50 mM MES, 0.5% glucose, 2 mM NaPO_4_, 100 μM Acetosyringone) before being mechanically infused (5 ml syringe) into multiple individual tobacco (*N. tobacum*, cv. SR1) leaves (2–4 leaves per test construct). Assays were performed in groups of 4–8 constructs and the resulting luciferase activities (both FiLUC and RiLUC) determined after 3–4 days using an *in vivo *floating leaf-disk assay developed for this enhancer screen [45]. Test constructs were assayed from 1 to 6 times, with each assay consisting of 2–4 disks (3 mm diameter) per infusion. The disks used *in vivo *assays were each measured for light production in separate wells of a white-walled 96 well microtiter plate (FLUOstar Optima luminometer^® ^from BMG Lab Technologies Inc.) and all elements that tested positive in the *in vivo *assay were subsequently confirmed using the *in vitro *dual-luciferase^® ^from Promega Corp (assays performed according to the manufacturers instructions, separate leaf disks from the same leaf infusions were used for the *in vivo *assays). Each test group included an infusion containing the 35S min construct (lacking any viral test element). In order to compare the various assay systems, all activities were normalized to the activity of the 35S min construct included within each assay set (35S min activity arbitrarily set to 1.0).

### Determining DNA sequence element frequency in viral and *Arabidopsis *databases

Since the element enrichment approach works best when applied to relatively short, core consensus sequences [31], database searches were limited to those viral enhancers that displayed a clear core consensus (CLE, BCTV DR19/30, CT-rich, Figure 3). Results from the viral enhancer searches were compared to values obtained using previously reported plant promoter elements (DRE, ABRE-like, and G-box), and a short DNA sequence that is part of a highly conserved geminiviral replication loop stem sequence (CGCGNCCA) that is identical in base composition and length to the CLE consensus (Table 1). The short sequence elements were each tested for their frequency of occurrence within a set of DNA sequence databases. One database consists of all entries for geminiviruses plus nanoviruses ([36], as of May, 2004) and all others are from the *A. thaliana *genomic sequence at the TAIR, PatMatch web site [32]. The geminivirus/nanovirus BLAST searches were set for short exact matches (the statistical significance threshold set to 1000 and word size set at the element's length), returning the number of occurrences of exact matches for the full length element within the database. The TAIR PatMatch searches (default settings: Max hits, 7500; both strands; mismatch = 0; minimum hits/seq = 1; maximum hits/seq = 100) were performed against sub-datasets representing *Arabidopsis *coding sequences {"GI CDS (- introns, - UTRs)"}, and various lengths of upstream regions {"Locus Upstream Sequences", -1 to -500, -1 to -1000 and -1 to -3000}. Results from the -500 search were subtracted from the -1000 results, to generate hits from -501 to -1000 and -1000 results subtracted from the -3000 data to calculate hits from -1001 to -3000. In order to allow direct comparison between searches in different databases, using sequence elements of differing length and base composition, the number of database hits was corrected for the size of the database (number of hits divided by the database size in mega-base pairs {Mbp}) and base composition (hits/Mbp divided by the predicted number of hits per Mbp using upon the element sequence and base composition of each search database). The dataset base compositions were determined from downloaded sequence files and are: *A. thaliana *CDS: A/T = 55.8%, G/C = 44.2%; *A. thaliana *upstream (-1 to -500): A/T = 67.43%, G/C = 32.57%; *A. thaliana *upstream (-501 to -1000): A/T = 66.24%, G/C = 33.76%; viral: A/T = 56.2%, G/C = 43.8%. The resulting frequency of occurrence is a corrected number of hits per mega-base pairs (cHits/Mbp). For ease of comparison between elements, all of the cHits/Mbp values have been normalized to the corresponding cHits/Mbp number from the *A. thaliana *CDS database (set arbitrarily to 1.0). Correction of the element's frequency using the calculated random probability of occurrence does not account for the possible impacted by intrinsic base-order bias that may occur within each sequence database, specifically the coding region database. These biases can potentially shift cHits/Mbp numbers markedly from those calculated using simple random base composition frequencies. To help confirm the significance of any observed enhancement in an elements frequency, mean cHits/Mbp values for 18 randomly generated sequences that match each test sequence for base composition and length were determined to provide a baseline value for comparison to that of the test element (shown in parenthesis, Table 1). A total of 18 sequences were used to produce the reported baseline as mean cHits/Mbp values were found to routinely level off at n values of between 8–12 random sequences examined (data not shown).

## Competing interests

A patent application is being considered for synthetic plant promoters containing some of the elements described in this article.

## Disclaimer

Mention of trade names or commercial products in this article is solely for the purpose of providing specific information and does not imply recommendation or endorsement by the U.S. Department of Agriculture.

## Authors' contributions

JV conceived of the study, participated in its design and coordination and drafted the manuscript. KM performed much of the search for short repeats within viral sequences and contributed to development of protoplast-based reporter gene assays. CIC generated and tested all the elements examined and developed the *in vivo *assay used to quantify enhancer activity. All authors read and approved the final manuscript.

## Supplementary Material

Additional File 1 Excel worksheet listing viral elements that fail to enhance expression Click here for file
